# Plasma Level of Adrenomedullin Is Influenced by a Single Nucleotide Polymorphism in the Adiponectin Gene

**DOI:** 10.1371/journal.pone.0070335

**Published:** 2013-08-01

**Authors:** Hoi Kin Wong, Kwok Leung Ong, Raymond Y. H. Leung, Tommy T. Cheung, Aimin Xu, Tai Hing Lam, Karen S. L. Lam, Bernard M. Y. Cheung

**Affiliations:** 1 Department of Medicine, University of Hong Kong, Hong Kong; 2 Centre for Vascular Research, University of New South Wales, Sydney, New South Wales, Australia; 3 Department of Community Medicine and School of Public Health, University of Hong Kong, Hong Kong; University of North Carolina at Chapel Hill, United States of America

## Abstract

**Objective:**

Adrenomedullin (ADM) and adiponectin are both involved in inflammation and cardiovascular diseases. The plasma levels of these peptides are influenced by single nucleotide polymorphisms (SNPs) in the *ADM* and *ADIPOQ* genes respectively. There is some evidence that ADM may regulate adiponectin gene expression, but whether adiponectin can regulate ADM expression is unclear, and was therefore investigated.

**Methods:**

Plasma ADM level was measured in 476 subjects in the Hong Kong Cardiovascular Risk Factor Prevalence Study-2 (CRISPS2). We genotyped them for 2 *ADIPOQ* SNPs that are known to be associated with plasma adiponectin level.

**Results:**

The minor allele frequencies of *ADIPOQ* SNPs rs182052 and rs12495941 were 40.6% and 42.2% respectively. Plasma ADM level was significantly associated with rs182052 after adjusting for age and sex (β = 0.104, P = 0.023) but not with rs12495941 (β = 0.071, P = 0.120). In multivariate analysis, plasma ADM level increased with the number of minor alleles of rs182052 (P = 0.013). Compared to subjects with GG genotype, subjects with AA genotype had 17.7% higher plasma ADM level (95% CI: 3.6%–33.7%). Subgroup analysis revealed that the association was significant in diabetic patients (β = 0.344, P = 0.001) but not in non-diabetic subjects.

**Conclusion:**

Plasma ADM level is related to SNP rs182052 in the *ADIPOQ* gene. Our findings provide new evidence of the interplay between these two important peptides in cardiovascular disease and diabetes. Knowing the genotype may help to refine the interpretation of these biomarkers.

## Introduction

Adipose tissue is now recognized as a key endocrine organ which secretes hormones known as adipokines [1]. One of these is adiponectin, which has insulin-sensitizing effect by promoting lipid β-oxidation and hepatic gluconeogenesis [2]. It is thought to counteract insulin resistance and its serum level is decreased in obesity and type 2 diabetes [3].

Recently, adrenomedullin (ADM) has also been recognized as an adipokine [4]. ADM is a 52-amino acid peptide and its plasma level is elevated in many clinical conditions including hypertension, septic shock, renal failure and in type 2 diabetes [5], [6]. While plasma ADM level is elevated in these pathological conditions, it is also partly influenced by genetic variation. Previously our group has reported that the plasma level of ADM is associated with its single nucleotide polymorphism (SNP) (rs4910118) [7], and SNP in the interleukin-6 gene (rs17147230) [8]. In this study we hypothesized that plasma ADM level might also be affected by genetic variations in the gene encoding adiponectin. This gene, known as the *ADIPOQ* gene, is located in the chromosome 3q27 region. Previous studies have shown that adiponectin SNPs are associated with the components of the metabolic syndrome [9],[10]. Recently, our group has also confirmed that *ADIPOQ* gene variants are associated with hypertension and adiponectin levels in a Chinese sample [11].

Since ADM and adiponectin are both secreted from adipose tissues and are related to the metabolic syndrome, they can be related in other ways. A previous study has shown an association between plasma adiponectin level and mid-region pro-adrenomedullin level (MR-proADM) [12]. It has also been reported that ADM decreases *ADIPOQ* gene expression in epididymal fat [13]. Whether the *ADIPOQ* gene may affect ADM level is not clear. Therefore we aimed to investigate the association between *ADIPOQ* SNPs and plasma ADM level.

## Methods

### Subjects

The subjects included came from the Hong Kong Cardiovascular Risk Factor Prevalence Study (CRISPS), which is a cohort study of cardiovascular risk factors in Hong Kong Chinese [14], [15]. Initially, 2895 subjects were recruited in 1995–1996 (CRISPS1), and 1944 of them were followed up in 2000–2004 (CRISPS2) after a median interval of 6.4 years. The study protocol was approved by the Ethics Committee of the University of Hong Kong and the Institutional Review Board of the Hong Kong West Cluster of Hospitals. All subjects had given written consents. Plasma ADM levels were measured in 476 subjects who were randomly chosen from the cohort of 1944 subjects [7], [8].

### SNP Selection

Tagging SNPs from the *ADIPOQ* gene were selected from the HapMap Han Chinese (phase II data, release 24). Using the HapMap data, 14 *ADIPOQ* SNPs were identified in our previous study [11], which were located within 5-kb region upstream to 2-kb downstream of the gene (position 188,038,157–188,060,946) with *r^2^*≥0.9 and minor allele frequency (MAF) ≥0.05. Four of the SNPs were found to be significantly associated with plasma adiponectin level. The association was the strongest in two SNPs, rs182052 and rs12495941 (P<0.0001) [11]. Therefore in this analysis we chose to focus on these two SNPs to investigate their correlation with plasma ADM level. The SNPs were genotyped using the MassARRAY system (Sequenom, San Diego, CA, USA) with the iPLEX assay in the Genome Research Centre, The University of Hong Kong.

### Plasma ADM Levels and Other Variables of Interest

Plasma samples were extracted and ADM levels were measured by radioimmunoassay (RIA) using a method adapted from our laboratory previously [16]. The immunoreactivity of plasma ADM was measured by commercially available kits from Peninsula Laboratories (Belmont, CA, USA) in 235 subjects and Phoenix Pharmaceuticals (Burlingame, CA, USA) in 241 subjects, using the same protocol with internal controls to ensure comparability. Other clinical parameters such as glucose, insulin and lipid profiles were measured as previously described [14], [15], [17]. Serum IL-6, soluble tumor necrosis factor alpha receptor 2 (TNF-α R2), high-sensitivity C-reactive protein (hsCRP) and adiponectin were measured from stored samples collected at CRISPS2 with standard protocols which were previously described [18]. Regular drinking was defined as alcohol consumption for at least once a week. Current smoking was defined as smoking at least one cigarette every week. Regular exercise was defined as having exercise for at least once a week in the past month. Diabetes is classified according to the World Health Organization 1998 diagnostic criteria [19]. In the multivariate analysis, the associations were adjusted using different models. Since adiponectin is associated with obesity, insulin resistance, hypertension, inflammation and non-alcoholic fatty liver diseases [20], [21], therefore in model 3 we adjusted for the markers related to these conditions. Adiponectin level is also associated with lifestyle factors such as smoking and physical exercise [22], so in model 4 we further adjusted for 3 lifestyle factors (status of regular smoking, alcohol drinking, and frequency of exercise).

### Statistical Analysis

Regression analysis was performed using SPSS 19.0 for Windows (SPSS Inc, Chicago, IL, USA). Normally distributed variables were expressed as means ± SD, while those with skewed distributions were natural log-transformed and expressed as geometric means (95% confidence interval). For variables that were highly correlated such as BMI and waist circumference, only one was allowed in the regression model at any one time. Multiple linear regression models were used to estimate the regression coefficients. Correction for multiple testing was performed using permutation test with the simulation repeated 1,000 times. Linkage disequilibrium was assessed using PLINK software (version 1.0.7) [23]. The P value for interaction was estimated by including a multiplicative interaction term in the multivariate model after adjusting for the main effects of the covariates. Clinical characteristics of subjects with or without diabetes mellitus were compared using independent t-test or Chi Square test.

## Results

### Genotyping of ADIPOQ SNPs and Subject Characteristics

The MAFs of rs182052 and rs12495941 were 40.6% and 42.2% respectively. Both SNPs were in Hardy Weinberg equilibrium (P = 0.48 and 0.45 for rs182052 and rs12495941 respectively). The pairwise linkage disequilibrium (*r^2^*) was 0.429.

Table 1 shows the characteristics of the 476 subjects with plasma ADM levels according to the genotypes of rs182052 and rs12495941. Both rs182052 and rs12495941 were significantly associated with adiponectin level in this cohort (P≤0.002). The minor allele of SNP rs182052 was associated with lower adiponectin levels while that of rs12495941 was associated with higher adiponectin levels. Also both SNPs were associated with LDL-cholesterol. The minor allele of SNP rs182052 was associated with higher LDL-cholesterol levels (P = 0.01) while the minor allele of rs12495941 was associated with lower LDL-cholesterol levels (P = 0.008). There is no significant interaction of plasma ADM levels with sex (P>0.05), so the association was analyzed in the whole population.

**Table 1:** Subject characteristics according to different genotypes of rs182052 and rs12495941.

Characteristics	rs182052				rs12495941			
	GG	GA	AA	P for trend	GG	GT	TT	P for trend
n	164	238	74	–	163	224	89	–
Age (years)	50.0±10.7	51.6±10.7	50.8±11.0	0.344	51.3±11.3	51.4±10.5	49.0±10.2	0.173
Women (%)	46.3	54.2	47.3	0.556	50.3	53.1	43.8	0.463
BMI (kg/m^2^)	23.7±3.2	23.8±3.7	24.5±3.4	0.150	24.2±3.7	23.6±3.4	23.9±3.2	0.362
Waist circumference (cm)	79.1±9.8	79.5±10.3	81.7±10.1	0.050	80.9±10.3	79.1±10.2	79.1±9.6	0.053
SBP (mmHg)	122.1±17.6	123.5±18.6	121.8±17.7	0.792	123.0±17.2	123.1±18.7	121.4±18.4	0.966
DBP (mmHg)	76.5±10.4	76.6±10.7	77.7±9.0	0.474	77.1±9.9	76.3±10.8	77.1±10.2	0.880
Triglycerides (mmol/L)*	1.20 (1.04–1.36)	1.16 (1.05–1.27)	1.24 (1.06–1.43)	0.850	1.21 (1.09–1.33)	1.16 (1.04–1.28)	1.22 (0.98–1.46)	0.973
HDL cholesterol (mmol/L)	1.38±0.42	1.39±0.37	1.31±0.36	0.182	1.34±0.37	1.40±0.38	1.35±0.42	0.386
LDL cholesterol (mmol/L)	3.13±0.84	3.30±0.84	3.42±0.82	0.010	3.36±0.86	3.26±0.81	3.06±0.84	0.008
Fasting glucose (mmol/L)*	5.22 (4.99–5.46)	5.19 (5.06–5.32)	5.48 (5.06–5.89)	0.234	5.37 (5.14–5.59)	5.18 (5.04–5.33)	5.17 (4.82–5.53)	0.154
Glucose 2 hours after OGTT (mmol/L)*	6.67 (6.20–7.14)	6.89 (6.51–7.26)	7.05 (6.20–7.90)	0.368	6.95 (6.45–7.46)	6.67 (6.31–7.02)	7.05 (6.30–7.80)	0.714
HOMA-IR*	1.70 (1.39–2.01)	1.68 (1.46–1.90)	1.93 (1.56–2.30)	0.308	1.81 (1.56–2.07)	1.69 (1.42–1.97)	1.64 (1.40–1.87)	0.237
Adiponectin (mg/L)*	7.74 (6.88–8.61)	6.82 (6.22–7.42)	5.50 (4.59–6.41)	<0.001	6.26 (5.59–6.93)	7.04 (6.35–7.74)	7.77 (6.70–8.85)	0.002
Fibrinogen (g/L)	2.94±0.56	2.90±0.52	2.96±0.51	0.822	2.89±0.50	2.93±0.54	2.95±0.58	0.157
CRP (mg/L)*	0.56 (0.43–0.68)	0.57 (0.41–0.72)	0.55 (0.37–0.73)	0.873	0.54 (0.34–0.74)	0.58 (0.48–0.69)	0.55 (0.36–0.73)	0.661
GGT (U/L)*	24.88(19.60–30.16)	22.60(20.42–24.78)	26.09(18.80–33.37)	0.940	24.82(20.32–29.31)	23.09(20.56–25.62)	24.27(16.56–31.98)	0.454
ALP (U/L)*	68.57(65.39–71.74)	67.91(65.45–70.36)	68.18(63.72–72.64)	0.683	67.68(64.73–70.62)	67.63(64.91–70.36)	70.51(66.68–74.34)	0.235
IL-6 (pg/L)*	0.46 (0.34–0.58)	0.48 (0.40–0.55)	0.49 (0.37–0.60)	0.565	0.50 (0.41–0.60)	0.43 (0.37–0.49)	0.52 (0.31–0.72)	0.893
TNF-α R2 (pg/mL)*	1911.21 (1806.45–2015.97)	1846.59 (1779.00–1914.18)	1863.67 (1764.86–1962.48)	0.198	1834.20 (1770.59–1897.81)	1893.34 (1813.97–1972.71)	1884.71 (1732.19–2037.22)	0.163
ADM (pmol/L)*	11.09(10.35–11.83)	11.38(10.66–12.09)	13.03(11.78–14.29)	0.023	11.05(10.43–11.68)	12.34 (11.51–13.17)	11.18 (9.29–13.07)	0.097
Current smoking (%)	21.1	17.2	18.9	0.938	17.2	17.9	24.7	0.310
Regular drinking (%)	11.2	10.4	8.2	0.642	10.7	10.1	10.2	0.693
Regular exercise (%)	30.2	29.1	28.2	0.630	30.8	29.4	26.7	0.653

Data are expressed as mean ± SD unless otherwise specified.

P values are adjusted for age and sex (except for age and sex respectively).

* Variables with skewed distribution are natural log-transformed and expressed as geometric mean (95% confidence interval).

Abbreviations: BMI, body mass index; SBP, systolic blood pressure; DBP, diastolic blood pressure; HDL, high-density lipoprotein; LDL, low-density lipoprotein; OGTT, oral glucose tolerance test; HOMA-IR, homeostatic model assessment of insulin resistance index; ADM, adrenomedullin; hsCRP, high-sensitivity C-reactive protein; GGT, gamma-glutamyltransferase; ALP, alkaline phosphatase; IL-6, interleukin-6; TNF-α R2, soluble tumor necrosis factor alpha receptor 2; ADM, adrenomedullin.

### Association of ADIPOQ SNPs with Plasma ADM Level

**Figure 1:**
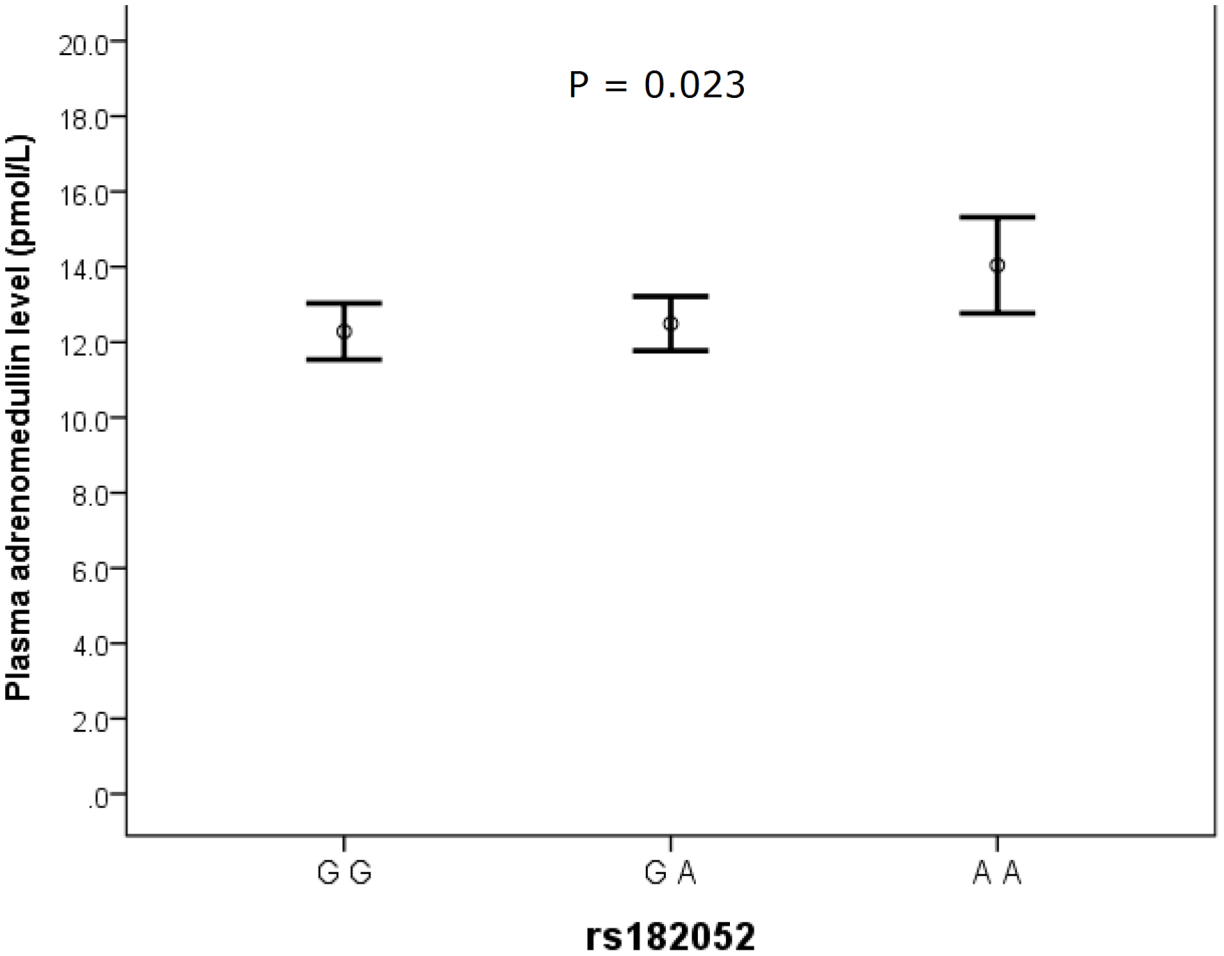
A diagram showing plasma ADM levels with different genotypes in rs182052. The error bars represent mean ±95% CI. P values were calculated using natural log-transformed levels after adjusting for age and sex.

Figure 1 shows the plasma ADM levels in subjects with different genotypes in rs182052. There was a significant association between number of minor allele of rs182052 and plasma ADM levels (β = 0.104, P = 0.023 after adjusting for age and sex). The association remains significant after adjusting for different biochemical markers (model 3) and lifestyle factors (model 4), suggesting that the association was independent of these covariates (Tables 2 and 3). However, rs12495941 was not significantly associated with ADM level (β = 0.076, P = 0.097 after adjusting for age and sex).

**Table 2:** Multivariate analysis of rs182052 with plasma ADM level (ln-transformed).

Model		Model 1(n = 476)	Model 2(n = 476)	Model 3(n = 424)	Model 4(n = 398)
Additive Model	β*	0.100	0.104	0.118	0.114
	*r^2^*	0.026	0.027	0.046	0.031
	Pˆ	0.029	0.023	0.022	0.026
Genotypic Model (with reference to GG genotype)					
AA	B** (SE)	0.161 (0.065)	0.163 (0.065)	0.181 (0.069)	0.175 (0.073)
	P	0.014	0.013	0.009	0.018
AG	B** (SE)	0.026 (0.047)	0.033 (0.048)	0.033 (0.050)	0.040 (0.053)
	P	0.583	0.492	0.509	0.459

* Standardized regression coefficient is shown.

** Unstandardized regression coefficient is shown.

∧ P values remain significant after permutation test for 1000 times (P<0.05).

Model 1: Unadjusted model.

Model 2: Adjusted for age and sex only.

Model 3: Further adjusted for biochemical parameters including waist circumference, LDL cholesterol, SBP, fibrinogen and natural log of triglycerides, HOMA-IR, fasting glucose level, hsCRP, adiponectin, interleukin-6, TNF-α R2, ALP and GGT.

Model 4: Further adjusted for lifestyle factors such as regular drinking, smoking and exercise.

**Table 3:** Association of covariates with plasma ADM level (ln-transformed).

Variables	β**	P value
rs182052	0.114	0.026
Age (years)	−0.059	0.355
Sex	−0.055	0.405
Waist circumference (cm)	0.101	0.163
SBP (mmHg)	−0.023	0.698
Triglycerides (mmol/L)*	−0.015	0.807
LDL cholesterol (mmol/L)	−0.046	0.390
Fasting glucose (mmol/L)*	−0.006	0.923
HOMA-IR*	−0.023	0.757
Adiponectin (mg/L)*	0.055	0.351
Fibrinogen (g/L)	0.042	0.483
CRP (mg/L)*	0.073	0.238
GGT (U/L)*	0.040	0.507
ALP (U/L)*	−0.111	0.070
IL-6 (pg/L)*	−0.098	0.074
TNF-α R2 (pg/mL)*	−0.030	0.603
Current smoking (%)	0.005	0.931
Regular drinking (%)	−0.032	0.550
Regular exercise (%)	<0.001	0.996

* Variables with skewed distribution were natural log transformed before analysis.

** Standardized regression coefficient beta was shown.

In a separate analysis, A*DIPOQ* SNPs were used as categorical variables in multivariate analysis (Table 2). Presence of two minor A alleles in rs182052 resulted in a 17.7% increase (95% CI: 3.6%, 33.7%) in plasma ADM level (P = 0.013). The increase in plasma ADM level was more significant after adjusting for other clinical parameters (20.0%, 95% CI: 4.8%–37.3%, P = 0.009).

Table S1 shows the subject characteristics according to diabetes status. There was a significant interaction between the minor allele of rs182052 and diabetes status after adjusting for their main effects (P = 0.001). The association between rs182052 and ADM levels was more significant in subjects with diabetes mellitus (β = 0.344, P = 0.001 after adjusting for age and sex). 12.3% of the variation in ADM level could be explained by the presence of the minor allele of rs182052 in diabetic patients after adjusting for age and sex (Table S2). In contrast, the association of the minor allele of rs12495941 with ADM level was weaker and not significant in non-diabetic subjects (β = 0.027, P = 0.596 after adjusting for age and sex).

## Discussion

A negative association between *IL6* SNP and plasma ADM levels has been demonstrated in our previous study [8]. The present report was the first to highlight an association between ADM and adiponectin in human. Since both IL-6 and adiponectin take part in inflammation, the associations of plasma ADM levels with *IL6* and *ADIPOQ* SNPs suggests that ADM levels are regulated by inflammatory factors and its elevation in diabetes and cardiovascular diseases could be due to inflammation that is known to occur in these diseases.

ADM and adiponectin are both hormones with well-recognized roles in the cardiovascular disease and diabetes. ADM is a vasodilatory peptide that has an important role in the cardiovascular system in health and in disease states. Plasma ADM level is elevated in hypertension, heart failure, acute myocardial infarction, atherosclerosis and type 2 diabetes [24], [25], [26]. The biological properties of ADM such as vasodilation, hypotension and anti-oxidation suggest that the elevated level may act as a compensatory response to cardiovascular diseases. Also a stable form of ADM, MR-proADM could act as a prognostic marker to predict mortality and survival of patients with heart failure and coronary heart diseases [27]. One study reports that it is even superior to CRP and adiponectin in predicting future cardiovascular events [28].

In contrast, adiponectin is an adipokine the level of which is lowered in cardiometabolic diseases. Decreased level is observed in type 2 diabetes, the metabolic syndrome [20], endothelial dysfunction and coronary heart disease [29], [30]. Adiponectin has cardiovascular protective effects [31]. It protects against endothelial dysfunction and hypertension, and directly acts on cardiomyocytes to protect the heart from ischaemic injury and hypertrophy [32], [33]. Given the important roles of ADM and adiponectin in cardiovascular diseases, knowing the interactions between these two peptides may help to refine the interpretation of the levels of these biomarkers, and may even suggest new therapeutic approaches in combating these diseases.

**Figure 2:**
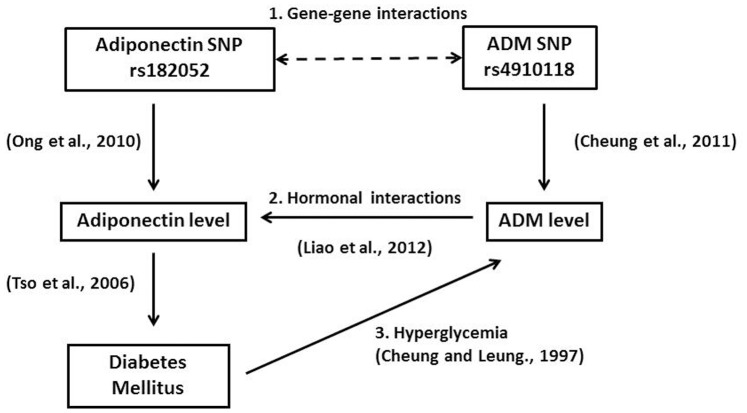
A schematic diagram summarizing the interplay between adiponectin and ADM. Adiponectin and ADM could regulate each other in 3 ways: 1. Gene-gene interactions from the respective SNPs; 2. ADM could increase adiponectin gene expression and secretion [13. Liao et al., 2012]; 3. In type 2 diabetes, plasma adiponectin level tends to be lower [36. Tso et al., 2006], while plasma ADM level tends to be higher [6. Cheung and Leung, 1997).

Various genetic association studies have demonstrated that the *ADIPOQ* gene is associated with plasma adiponectin level [34], [35]. Our group has reported that the minor allele of rs182052 was significantly associated with lower adiponectin level while the minor allele of rs12495941 was significantly associated with higher level [11]. However only the minor allele of rs182052 was significantly correlated with plasma ADM level and the association was independent of plasma adiponectin level (Table 3). Figure 2 summarizes the interplay between adiponectin and ADM based on findings from this study and previous studies. Adiponectin and ADM could regulate each other in 3 ways: 1. The adiponectin SNP rs182052 could interact with *ADM* SNP since we previously showed that *ADM* SNPs could influence plasma ADM level [7]; 2. ADM could directly increase adiponectin gene expression and secretion in vitro [13]; 3. In type 2 diabetes, plasma adiponectin level tends to be lower [36], while plasma ADM level tends to be higher [6].

Although both rs182052 and rs12495941 are located in intron 1 of *ADIPOQ*, rs12495941 is not involved in any putative transcription binding site, whereas presence of the minor allele of rs182052 results in the loss of Sp1 binding site and the gain of a CCAAT/enhancer binding protein (C/EBP) β binding site, both of which promotes adipocyte differentiation [37]. Since ADM is highly expressed in differentiated adipocytes [38], further studies are required to show whether an increase in ADM is due to enhanced expression from differentiated adipocytes.

Previous studies have demonstrated the roles of ADM and adiponectin in inflammation. ADM expression is increased in inflammation prior to administration of endotoxin [39]. It has both pro-inflammatory and anti-inflammatory actions in macrophages and modulates cytokine response [40]. It can also stimulate release of inflammatory mediators like migratory inhibitory factor (MIF) and IL-1β while suppressing tumor-necrosis factor alpha (TNF-α). In contrast, adiponectin exhibits anti-inflammatory effects [41]. It inhibits TNF-α production and NF-κB activation, as well as stimulates production of anti-inflammatory cytokines [42]. Adiponectin also inhibits macrophage activation and both leptin- and lipopolysaccharide–induced production of pro-inflammatory cytokines in macrophages [43]. A recent study suggested that ADM increased IL-6 expression and the latter decreased adiponectin expression. Hence, IL-6 might mediate the inhibitory effect of ADM on adiponectin expression [13]. However, in the present study the association between adiponectin SNP and ADM level was not confounded by IL-6. The interaction between adiponectin and ADM in inflammation deserves further investigation.

The SNP rs182052 was reported to be significantly associated with type 2 diabetes in an Asian sample [44]. Although the role of ADM in diabetes has not been well established, elevated plasma ADM levels were observed in diabetic patients [37]. The stratified analysis in the present study further showed that diabetes could influence the association between rs182052 and ADM level, as a greater effect size (β = 0.344) was demonstrated in diabetic patients. However the limitation of our study was the small sample size of diabetic patients with low statistical power and precision. Further studies with a larger sample size are needed to confirm this finding.

In the present study, carriers of minor allele A at rs182052 had significantly higher LDL-cholesterol level while carriers of minor allele T at rs12495941 had significantly lower LDL-cholesterol level (P≤0.01) (Table 1). A recent study reported that serum adiponectin may be inversely correlated with LDL [45]. Since the minor allele of rs182052 is negatively associated with adiponectin, while minor allele of rs12495941 is positively associated with adiponectin, this could account for the opposite trend in the two SNPs with LDL. It is well known that high LDL level is associated with higher risk of atherosclerosis and coronary artery disease (CAD) [46]. Hence the former group of subjects had potentially higher risk of atherosclerosis and CAD.

In conclusion, this study has demonstrated a significant association between *ADIPOQ* SNP rs182052 and plasma ADM level. This enhances our understanding of the regulation of ADM level and points to the close interplay between these two hormones that could be very important in cardiovascular diseases. Further studies are necessary to elucidate the function of the *ADIPOQ* SNP and how it could influence ADM level.

## Supporting Information

Table S1 Subject characteristics according to diabetes mellitus status Characteristics are compared by independent t-test for continuous variable and Chi Square test for categorical variables. Data are expressed as mean ± SD unless otherwise specified. *Variables with skewed distribution are natural log-transformed and expressed as geometric mean (95% confidence interval). Abbreviations: BMI, body mass index; SBP, systolic blood pressure; DBP, diastolic blood pressure; HDL, high-density lipoprotein; LDL, low-density lipoprotein; OGTT, oral glucose tolerance test; HOMA-IR, homeostatic model assessment of insulin resistance index; ADM, adrenomedullin; hsCRP, high-sensitivity C-reactive protein; GGT, gamma-glutamyltransferase; ALP, alkaline phosphatase; IL-6, interleukin-6; TNF-α R2, soluble tumor necrosis factor-alpha receptor 2.
(DOC)

Table S2 Association of number of minor alleles in rs182052 with plasma ADM level. *Standardized regression coefficient is shown. Model 1: Unadjusted model. Model 2: Adjusted for age and sex only. Model 3: Further adjusted for biochemical parameters including waist circumference, LDL cholesterol, SBP, fibrinogen and natural log of triglycerides, HOMA-IR, fasting glucose level, hsCRP, adiponectin, interleukin-6, TNF-α R2, ALP and GGT. Model 4: Further adjusted for lifestyle factors such as regular drinking, smoking and exercise.
(DOC)
