# Supplementary material for: Plasma Level of Adrenomedullin Is Influenced by a Single Nucleotide Polymorphism in the Adiponectin Gene
Source: PLoS One. 2013 Aug 1;8(8):e70335. doi: 10.1371/journal.pone.0070335 (PMC3731362; doi:10.1371/journal.pone.0070335)
Supplement: Table S1 — Subject characteristics according to diabetes mellitus status Characteristics are compared by independent t-test for continuous variable and Chi Square test for categorical variables. Data are expressed as mean ± SD unless otherwise specified. *Variables with skewed distribution are natural log-transformed and expressed as geometric mean (95% confidence interval). Abbreviations: BMI, body mass index; SBP, systolic blood pressure; DBP, diastolic blood pressure; HDL, high-density lipoprotein; LDL, low-density lipoprotein; OGTT, oral glucose tolerance test; HOMA-IR, homeostatic model assessment of insulin resistance index; ADM, adrenomedullin; hsCRP, high-sensitivity C-reactive protein; GGT, gamma-glutamyltransferase; ALP, alkaline phosphatase; IL-6, interleukin-6; TNF-α R2, soluble tumor necrosis factor-alpha receptor 2. (DOC) [file pone.0070335.s001.doc]

**Supplementary Tables**

Supplementary Table S1. Subject characteristics according to diabetes mellitus status

| Characteristics | Without DM | With DM | P value |
| --- | --- | --- | --- |
| n | 392 | 84 | - |
| Age (years) | 49.6 ± 10.2 | 57.0 ± 11.2 | <0.001 |
| Women (%) | 51.8 | 44.0 | 0.229 |
| BMI (kg/m^2^) | 23.6 ± 3.3 | 25.0 ± 4.2 | 0.001 |
| Waist circumference (cm) | 79.0 ± 9.8 | 83.1 ± 11.1 | 0.001 |
| SBP (mmHg) | 121.1 ± 17.4 | 130.4 ± 19.3 | <0.001 |
| DBP (mmHg) | 76.4 ± 10.1 | 78.4 ± 11.4 | 0.116 |
| Triglycerides (mmol/L)* | 1.16 (1.06-1.25) | 1.33 (1.16-1.50) | 0.027 |
| HDL cholesterol (mmol/L) | 1.40 ± 0.39 | 1.24 ± 0.34 | <0.001 |
| LDL cholesterol (mmol/L) | 3.24 ± 0.82 | 3.35 ± 0.90 | 0.255 |
| Fasting glucose (mmol/L)* | 5.01 (4.93-5.10) | 6.47 (6.00-6.95) | <0.001 |
| Glucose 2 hours after OGTT (mmol/L)* | 6.46 (6.25-6.67) | 10.12 (8.85-11.39) | <0.001 |
| HOMA-IR* | 1.60 (1.43-1.76) | 2.45 (1.99-2.91) | <0.001 |
| Adiponectin (mg/L)* | 7.13 (6.62-7.64) | 5.86 (5.00-6.73) | 0.011 |
| Fibrinogen (g/L) | 2.90 ± 0.51 | 3.04 ± 0.61 | 0.030 |
| CRP (mg/L)* | 0.54 (0.46-0.61) | 0.68 (0.31-1.06) | 0.042 |
| GGT (U/L)* | 23.19 (20.41-25.97) | 27.44 (22.98-31.90) | 0.024 |
| ALP (U/L)* | 67.40 (65.50-69.30) | 71.88 (67.12-76.64) | 0.063 |
| IL-6 (pg/L)* | 0.46 (0.39-0.53) | 0.52 (0.41-0.61) | 0.363 |
| TNF-α R2 (pg/mL)* | 1847.28 (1795.62-1898.94) | 1987.49 (1823.13-2151.85) | 0.578 |
| Current smoking (%) | 18.4 | 21.4 | 0.540 |
| Regular drinking (%) | 10.7 | 8.5 | 0.691 |
| Regular Exercise (%) | 27.4 | 38.3 | 0.060 |
