# Supplementary material for: Plasma Level of Adrenomedullin Is Influenced by a Single Nucleotide Polymorphism in the Adiponectin Gene
Source: PLoS One. 2013 Aug 1;8(8):e70335. doi: 10.1371/journal.pone.0070335 (PMC3731362; doi:10.1371/journal.pone.0070335)
Supplement: Table S2 — Association of number of minor alleles in rs182052 with plasma ADM level. *Standardized regression coefficient is shown. Model 1: Unadjusted model. Model 2: Adjusted for age and sex only. Model 3: Further adjusted for biochemical parameters including waist circumference, LDL cholesterol, SBP, fibrinogen and natural log of triglycerides, HOMA-IR, fasting glucose level, hsCRP, adiponectin, interleukin-6, TNF-α R2, ALP and GGT. Model 4: Further adjusted for lifestyle factors such as regular drinking, smoking and exercise. (DOC) [file pone.0070335.s002.doc]

Supplementary Table S2. Association of number of minor alleles in rs182052 with plasma ADM level

|  | Without DM | | | | With DM | | | |
| --- | --- | --- | --- | --- | --- | --- | --- | --- |
|  | Model 1 | Model 2 | Model 3 | Model 4 | Model 1 | Model 2 | Model 3 | Model 4 |
| n | 392 | 392 | 347 | 326 | 84 | 84 | 77 | 72 |
| β* | 0.025 | 0.027 | 0.031 | 0.031 | 0.360 | 0.344 | 0.366 | 0.360 |
| *r^2^* | 0.018 | 0.016 | 0.055 | 0.040 | 0.123 | 0.123 | 0.109 | 0.060 |
| P | 0.612 | 0.596 | 0.566 | 0.590 | 0.001 | 0.001 | 0.005 | 0.013 |
